# Preoperative Platelet Distribution Width Represents a Novel Prognostic Biomarker in Patients With Nonmetastatic Renal Cell Carcinoma: A Retrospective Clinical Analysis

**DOI:** 10.3389/fonc.2022.845028

**Published:** 2022-03-31

**Authors:** Ruotao Xiao, Bin Yang, Cheng Liu, Lei Liu, Lulin Ma

**Affiliations:** Department of Urology, Peking University Third Hospital, Beijing, China

**Keywords:** platelet distribution width, biomarker, renal cell carcinoma, prognosis, risk stratification

## Abstract

**Purpose:**

The study aimed to explore the prognostic value of platelet distribution width (PDW) in patients with nonmetastatic renal cell carcinoma (RCC).

**Methods:**

We retrospective analyzed 706 patents with nonmetastatic RCC from January 2015 to December 2017. Clinicopathologic data and platelet indices were collected and analyzed by univariable and multivariable cox proportional hazard model. Progression-free survival (PFS) was analyzed using the Kaplan–Meier curve. Net reclassification improvement (NRI) and integrated discrimination improvement (IDI) were performed to evaluate the improvement of predictive accuracy.

**Results:**

Patients were divided into low PDW (N = 241, PDW ≤11.7%), intermediate PDW (N = 232, 11.7%< PDW ≤15.6%), and high PDW (N = 233, PDW >15.6%) groups according to the tertiles. Patients with low PDW were associated with more symptoms at presentation, larger tumor size, higher AJCC tumor stage, and more sarcomatoid differentiation. Besides, patients with low PDW had significantly shorter PFS compared to intermediate PDW and high PDW groups. On the multivariable model, AJCC tumor stage, nuclear grade, and PDW (either continuous or categorical variables) were independent factors correlated with PFS. The NRI and IDI showed adding PDW to SSIGN score improves its predictive accuracy related to 2-, 3-, and 4-year PFS.

**Conclusions:**

Low PDW was related to advanced clinicopathologic features and worse prognosis in patients with nonmetastatic RCC. Thus, PDW could serve as a novel biomarker for risk stratification in these patients when used pre-or postoperatively.

## Introduction

Renal cell carcinoma (RCC) is the third most frequent genitourinary cancer and accounts for 3–5% of all adult malignancies (1). Although surgery remains the most effective curative treatment for RCC, approximately 28% of patients with RCC develop metastatic disease after surgery (2). A predictive model of oncologic outcome is crucial which can help us to select high-risk patients for adjuvant therapeutic strategies in the future. Nowadays, several prognostic models have been established to predict the prognosis of localized RCC (3–5). However, most of the risk factors in these models rely on postoperative histopathologic parameters. Unfortunately, there is a lack of a mature biomarker for cancer diagnosis, monitor, and predicting prognosis. Thus, searching for a useful biomarker for prognostic stratification is of crucial importance.

Platelets have long been recognized as a key role in hemostasis and thrombosis. However, growing evidence suggests that the platelets can play an important role in the inflammatory response, influence the tumor microenvironment, and promote tumor growth (6, 7). Recently platelet indices such as platelet count (PLT), mean platelet volume (MPV), platelet distribution width (PDW) have been shown to carry the potential for prognostic information in several malignancies (8–11). PLT is widely recognized index reflecting the number of platelets in circulation system. Besides, MPV and PDW reflect the size and variation of platelets, and have been reported to be markers of platelet activation (12). In RCC patients, PLT and MPV have also been investigated as valuable factors for diagnosis and predicting oncologic outcomes (13, 14). However, the role of PDW especially in nonmetastatic RCC has not been investigated. It is presently unknown whether PDW could be routinely considered as a prognostic biomarker like other malignancies. Therefore, the study aimed to analyze the prognostic value of PDW in patients with nonmetastatic RCC.

## Materials and Methods

### Patients

We retrospectively analyzed the data of consecutive patients with RCC who underwent radical or partial nephrectomy at the Peking University Third Hospital between January 2015 and December 2017. Exclusion criteria were as follows: (1) metastatic, recurrent, and bilateral RCC; (2) combined with other malignancies; (3) those with a hematological disease, inflammatory disease, autoimmune disease; (4) a past history of splenectomy; (5) those using antiplatelet drugs within a week before blood collection; and (6) missing platelet data. Finally, 706 patients who met the inclusion criteria were further analyzed.

### Clinicopathologic Evaluation

Clinicopathologic parameters include age, sex, body mass index (BMI), comorbidities, symptoms, surgical methods, tumor size and side, tumor pathologic data (histologic subtype, nuclear grade, tumor stage, nuclear grade, necrosis, sarcomatoid and rheumatoid differentiations) were obtained through medical records. Tumor size was based on preoperative images and was defined as the greatest diameter in centimeters. The tumor stage was defined according to the 8th American Joint Committee on Cancer (AJCC) stage (15). The nuclear grade was defined mainly according to the Fuhrman grading system (16). SSIGN score was calculated according to the model reported by Frank et al. (4). Laboratory examination was performed within a week before surgery. Platelet indices were obtained and include PLT (×10^9^/L), MPV (fL), and PDW (%) through a complete blood count report.

### Follow Up

After surgery, patients were followed up and prognostic data were obtained through a clinic visit or by telephone. Patients were recommended to a follow-up for every 3 months in the first year, every 6 months in the next 2 years, and yearly thereafter. At each visit, laboratory examinations, X-rays, ultrasonic scans, or abdominal computed tomographs were performed. Disease progression was mainly evaluated by image examination or percutaneous biopsy and defined as any evidence for tumor recurrence at the surgical side or/and metastasis on the other side. Progression-free survival (PFS) was calculated from the date of surgery to disease progression.

### Statistical Analysis

Categorical variables were reported as whole numbers and proportions, and continuous variables were reported as medians with interquartile ranges (IQR). The analysis of variance (ANOVA) test or Kruskal–Wallis test were applied to compare continuous variables. The Chi-square test or Fisher exact test was applied to compare categorical variables. Univariable and multivariable Cox proportional hazard models were performed to identify the risk factors. The Kaplan–Meier curve was used to graphically illustrate the effect of PDW on PFS. Improvement in the predictive accuracy was assessed by calculating the integrated discrimination improvement (IDI) and the net reclassification improvement (NRI) (17). Higher IDI and NRI indicated greater risk discrimination and improved classification.

Statistical analysis was performed using SPSS (version 26, IBM Corp, Armonk, NY, USA) and R software (Version 4.0.3). All tests were two-side and p <0.05 was considered statistically significant.

## Results

We identified 706 patients with nonmetastatic RCC who underwent curative surgery in our study (**Table 1**). The median age at surgery was 58 years (IQR:49–64) and 483 (68.4%) patients were men. Approximately 77.5% (N = 547) of patients were asymptomatic at presentation. Approximately 53.4% (N = 377) of patients underwent nephron-sparing surgery (NSS) in our study cohort. A total of 465 (65.9%), 24 (3.4%), 206 (29.2%), 11 (1.6%) patients were in stages I, II, III, and IV respectively. According to the SSIGN score, patients with score 0–2, score 3–6, and score 7–11 were 502 (71.1%), 151 (21.4%), and 53 (7.5%) respectively. The median of PLT, MPV, and PDW were 213 × 10^9^/L (IQR: 179.75–251.25), 10.1 fL (IQR: 9.1–11), and 13.1% (IQR: 11.2–16.3) respectively. Patients were divided into low PDW (N = 241, PDW ≤11.7%), intermediate PDW (N = 232, 11.7%< PDW ≤15.6%), and high PDW (N = 233, PDW >15.6%) groups according to the tertiles. In our study cohort, patients with low PDW were associated with more symptom at presentation (P = 0.011), larger tumor size (P = 0.001), higher AJCC tumor stage (P <0.001), and more sarcomatoid differentiation (P = 0.017). While age, sex, and BMI failed to show significant association with PDW (**Table 2**).

**Table 1:** Descriptive characteristics of 706 patients treated with nephrectomy for nonmetastatic renal cell carcinoma.

Variable	All cohort (N = 706)
Age, year	58 (49–64)
Sex	
Male	483 (68.4)
Female	223 (31.6)
BMI, kg/m^2^	25.21 (23.11–27.34)
Symptom at presentation	
Absent	547 (77.5)
Present	159 (22.5)
Comorbidities	
Hypertension	279 (39.5)
Diabetes mellitus	115 (16.3)
Coronary disease	61 (8.6)
Surgical methods	
NSS	377 (53.4)
RN	329 (46.6)
Tumor size, cm	3.9 (2.7–5.6)
Tumor side	
left	343 (48.6)
Right	363 (51.4)
AJCC tumor stage	
I	465 (65.9)
II	24 (3.4)
III	206 (29.2)
IV	11 (1.6)
Histologic subtype	
ccRCC	615 (87.1)
Non-ccRCC	91 (12.9)
Nuclear grade	
I–II	515 (72.9)
III–IV	145 (20.5)
unknown	46 (6.5)
Necrosis	101 (14.3)
Sarcomatoid differentiation	12 (1.7)
Rheumatoid differentiation	12 (1.7)
SSIGN score	
0–2	502 (71.1)
3–6	151 (21.4)
7–11	53 (7.5)
Platelet indices	
PLT, ×10^9^/L	213 (179.75–251.25)
MPV, fL	10.1 (9.1–11)
PDW, %	13.1 (11.2–16.3)

BMI, body mass index; AJCC stage, American Joint Committee on Cancer stage; RN, radical nephrectomy; NSS, nephron sparing surgery; ccRCC, clear cell renal cell carcinoma; PLT, platelet count; MPV, mean platelet volume; PDW, platelet distribution width.

**Table 2:** Relationship between platelet distribution width and clinicopathologic characteristics.

Variable	Low PDW (N = 241, PDW ≤11.7%)	Intermediate PDW (N = 232, 11.7%< PDW ≤15.6%)	High PDW (N = 233, PDW >15.6%)	P
Age, year	59 (51–64)	55 (49–62.75)	58 (48–64)	0.281^£^
Sex				
Male	178 (73.9)	156 (67.2)	149 (63.9)	0.061^#^
Female	63 (26.1)	76 (32.8)	84 (36.1)	
BMI	25.22 (23.09–27.47)	25.39 (23.18–27.16)	25.15 (23.05–27.68)	0.687^£^
Symptom at presentation				
Absent	172 (71.4)	182 (78.4)	193 (82.8)	0.011^#^
Present	69 (28.6)	50 (21.6)	40 (17.2)	
Tumor size	4.1 (2.75–6.55)	3.95 (3–6)	3.5 (2.45–4.8)	0.001^&^
AJCC tumor stage				
I–II	143 (59.3)	164 (70.7)	182 (78.1)	<0.001^#^
III–IV	98 (40.7)	68 (29.3)	51 (21.9)	
Histologic subtype				
CCRCC	214 (88.8)	193 (83.2)	208 (89.3)	0.093^#^
Non-CCRCC	27 (11.2)	39 (16.8)	25 (10.7)	
Nuclear grade				
I–II	172 (75.4)	162 (75.3)	181 (83.4)	0.065^#^
III–IV	56 (24.6)	53 (24.7)	36 (16.6)	
Necrosis	37 (15.4)	35 (15.1)	29 (12.4)	0.610^#^
Sarcomatoid differentiation	9 (3.7)	2 (0.9)	1 (0.4)	0.017^*^
Rheumatoid differentiation	7 (2.9)	3 (1.3)	2 (0.9)	0.245^*^

£, The analysis of variance (ANOVA) test; &, Kruskal–Wallis test; #, Chi-square test; *, Fisher exact test.

**Figure 1:**
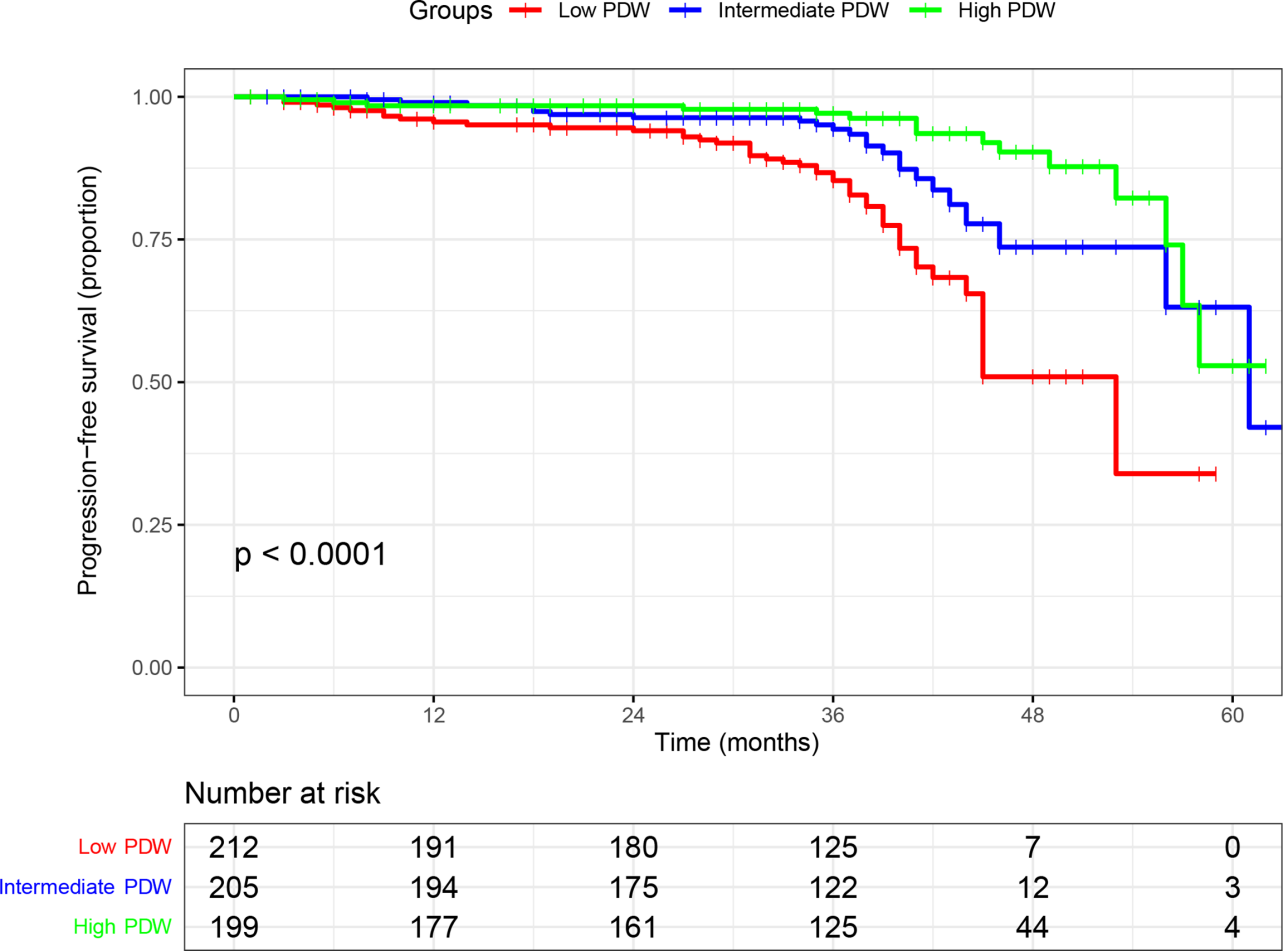
Kaplan–Meier curve of progression free survival in nonmetastatic renal cell carcinoma stratified according to platelet distribution width.

**Figure 2:**
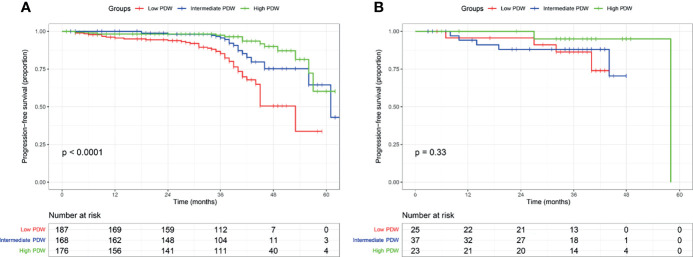
Kaplan–Meier curve of progression free survival in clear cell renal cell carcinoma **(A)** and non-clear cell renal cell carcinoma **(B)** stratified according to platelet distribution width.

In the cohort, 616 (87.3%) patients received follow-up with a median of 37 months (IQR: 33–41 months). A total of 85 (13.8%) patients experienced disease progression with a median PFS of 61 months (IQR: 46–not reached). In the Kaplan–Meier analysis, patients with low PDW were significantly associated with shorter PFS compared to intermediate and high PDW (**Figure 1**). In subgroup analysis, low PDW were also associated with shorter PFS compared to intermediate and high PDW in patients with ccRCC (**Figure 2A**). However, PDW were not significantly associated with PFS in patients with non-ccRCC (**Figure 2B**). On the univariable model, symptom at presentation (HR: 2.722, P <0.001), tumor size (HR: 1.139, P <0.001), AJCC tumor stage (HR: 7.345, P <0.001), nuclear grade (HR: 4.621, P <0.001), necrosis (HR: 4.066, P <0.001), sarcomatoid differentiation (HR: 8.927, P <0.001), rheumatoid differentiation (HR: 5.788, P <0.001), PLT (HR: 1.004, P = 0.002), PDW as continuous variable (HR: 0.826, P = 0.002), PDW as categorical variable (HR: 1.952 and 4.682, P = 0.047 and <0.001 for intermediate and low groups respectively) were associated with PFS. On the multivariable model, AJCC tumor stage (HR: 5.194, P <0.001), nuclear grade (HR: 2.355, P <0.001), PDW as a continuous variable (HR: 0.758, P <0.001), and PDW as a categorical variable (HR: 0.470 and 0.253, P = 0.004 and <0.001 for intermediate and high groups respectively) were independent factors associated with PFS (**Table 3**).

**Table 3:** Univariable and multivariable analysis predicting progress free survival of nonmetastatic renal cell carcinoma.

Variable	HR (95% Cl)	P	HR (95% Cl)	P
Age	1.019 (0.999–1.038)	0.059		
Sex				
Male	Ref			
Female	0.727 (0.450–1.173)	0.191		
Symptom at presentation				
Absent	Ref		Ref	
Present	2.722 (1.769–4.190)	<0.001	NA	0.197
BMI	0.991 (0.926–1.061)	0.793		
Tumor size	1.139 (1.092–1.189)	<0.001	NA	0.550
AJCC tumor stage				
I–II	Ref		Ref	
III–IV	7.345 (4.484–12.032)	<0.001	5.194 (3.073–8.780)	<0.001
Histologic subtype				
CCRCC	Ref			
Non-CCRCC	1.135 (0.601–2.144)	0.697		
Nuclear grade				
I–II	Ref		Ref	
III–IV	4.621 (2.971–7.189)	<0.001	2.355 (1.471–3.771)	<0.001
Necrosis				
Absent	Ref		Ref	
Present	4.066 (2.627–6.292)	<0.001	NA	0.456
Sarcomatoid				
Absent	Ref		Ref	
Present	8.927 (4.087–19.496)	<0.001	NA	0.062
Rheumatoid				
Absent	Ref		Ref	
Present	5.788 (2.512–13.336)	<0.001	NA	0.281
PLT	1.004 (1.002–1.007)	0.002	NA	0.182
MPV	0.976 (0.834–1.141)	0.759		
PDW	0.758 (0.690–0.832)	<0.001	0.758 (0.682–0.841)	<0.001
PDW^#^				
Low	Ref		Ref	
Intermediate	0.417 (0.251–0.692)	0.001	0.470 (0.280–0.790)	0.004
High	0.214 (0.117–0.389)	<0.001	0.253 (0.133–0.482)	<0.001

^#^, Separate model with the inclusion of PDW (categorical variable) and exclusion of PDW (continuous variable); NA, not available.

We established a modified SSIGN model by adding PDW to the conventional SSIGN model. The improvement of predictive accuracy between the modified SSIGN model and the conventional SSIGN model was analyzed using NRI and IDW values. The NRI values were 0.324 (95% Cl: 0.014–0.479, P = 0.04), 0.315 (95% Cl: 0.082–0.441, P = 0.02), 0.526 (95% Cl: 0.304–0.701, P = 0.01) for 2-, 3-, and 4-year PFS respectively. The IDI values were 0.028 (95% Cl: −0.007–0.069, P = 0.109), 0.052 (95% Cl: 0.011–0.094, P = 0.01), 0.145 (95% Cl: 0.069–0.190, P = 0.02) for 2-, 3-, and 4-year PFS respectively. These results suggested modified SSIGN model had a great improvement in predictive accuracy compared to the conventional SSIGN model.

## Discussion

In this study, we identified 706 consecutive patients with nonmetastatic RCC who underwent curative surgery in our institution and reported several noteworthy findings. Firstly, we observed that patients with low PDW have a strong and significant correlation with more advanced clinicopathologic features like more symptom at presentation, larger tumor size, advanced tumor stage, and sarcomatoid feature. Besides, we discovered patients with low PDW were associated with shorter PFS in the Kaplan–Meier curve compared to intermediate and high PDW groups, especially for patients with ccRCC. Furthermore, after adjusting for other clinicopathologic parameters, PDW has shown their independent prognostic value in predicting PFS in nonmetastatic RCC. Finally, adding PDW to the established SSIGN model improves its predictive accuracy in our study cohort. To our knowledge, our study was the first to explore the prognostic value of PDW in patients with nonmetastatic RCC.

Interestedly, as the most thoroughly studied platelet index, the previous studies have already demonstrated that PLT was a predictor for oncologic outcome in ovarian (18), lung (19), colorectal (20), gastric (21), breast cancers (22), and in RCC patients (23). Several studies have demonstrated that thrombocytosis had a strong correlation with adverse oncologic outcomes in RCC (13, 24). PLT has been added in the International Metastatic Renal Cell Carcinoma Database Consortium (IMDC) model to predict the prognosis of metastatic RCC (25). Other platelet indices like MPV have already shown a strong correlation with stage and prognosis (14, 26, 27), which suggested platelet indices should not be ignored as prognostic factors of worse survival in these patients. However, none of the studies have explored the usefulness of PDW in RCC populations. Our results suggested low PDW associated with more advanced clinicopathologic features and worse prognosis compared to intermediate and high PDW groups. More importantly, after controlling for PDW, PLT was no longer a risk factor predicting tumor progression. It is noteworthy that PDW represents the variation in platelet diameter, which could better reflect the activation of platelets rather than PLT (12). Basic experimental studies showed that tumorigenesis and metastasis can be promoted by activated platelets through a wide variety of crosstalk between platelets and cancer cells (6, 7): (1) Platelet activation releases growth factors and small molecules that facilitate tumor adhesion and extravasation, thereby supporting cancer cell transmigration and metastasis formation; (2) Platelet-tumor cell aggregates form and platelets protect circulating tumor cells (CTCs) from NK cell and TNF-a induced cell death. Based on the preclinical evidence and our findings, we speculate that PDW has the potential to be used as a novel biomarker to predict cancer metastasis in RCC populations.

Nevertheless, the data dealing with these subjects were conflicting among studies. Low PDW has proved to be a strong correlation with poor prognosis in lung (28), colon (29), hepatocellular (30), endometrial (31), and esophageal cancers (32), which is consistent with our findings. In the genitourinary tumors, low PDW has shown to be related to more advanced stages in bladder cancer (33). On the contrary, some studies have demonstrated high PDW was associated with adverse prognosis in breast (34), melanoma (35), ovary cancers (36) and skull base chordoma (37). It seems the prognostic value of PDW may be tissue-dependent and related to cancer-specific inflammation response. However, these studies were single-center retrospective analysis, which had different inclusion and exclusion criteria. We assumed this could be another reason for this conflict. In summary, the underline mechanism of PDW related to cancer metastasis is still unclear, which needed to clarify in further studies.

Several prognostic models existed to predict prognosis for nonmetastatic RCC, such as UISS model (3), SSIGN model (4), and Postoperative Karakiewicz’s Nomogram (5). Integrated prognostic factors included the presence of symptoms, ECOG-PS, TNM stage, Fuhrman grade, and tumor size. However, none of these integrated prognostic systems included any platelet indices. Although IMDC model has included PLT as a risk factor, it is mainly suitable for metastatic RCC (25). Since our study showed a strong association between PDW and oncologic outcomes in nonmetastatic RCC, we added PDW to SSIGN models and improve its predictive accuracy related to 2-, 3-, and 4-year PFS. However, it is undeniable that the results also need to be validated in an external cohort.

The current study has several limitations. Firstly, our study is a retrospective and single-center study, which inevitably existed confounding and information bias. As mentioned above, the prognostic value of PDW was conflicting among cancers. Thus, the results also need to be validated in an external multicenter cohort. Secondly, none of the study has investigated whether ethnic groups could affect platelet indices. Since the patients in our research were composed of Chinese populations, it also needs to be validated in other ethnic groups. Thirdly, although we have exhibited the prognostic value of PDW in patients with nonmetastatic RCC, the exact mechanism needs to be further elaborated in the basic experiment. However, our study is still worthy because we are the first study to investigate the prognostic value of PDW in patients with nonmetastatic RCC, and the results suggested that it can help us to do risk stratification when used pre-or postoperatively.

## Conclusion

Our findings suggested low PDW not only has a strong correlation with advanced clinicopathologic features but could predict worse prognosis in patients with nonmetastatic RCC. Thus, PDW could be considered as a promising biomarker for risk stratification in these patients when used pre-and postoperatively.

## Data Availability Statement

The raw data supporting the conclusions of this article will be made available by the authors, without undue reservation.

## Ethics Statement

The studies involving human participants were reviewed and approved by Peking University Third Hospital Medical Science Research Ethics Committee (IRB:00006761). The ethics committee waived the requirement of written informed consent for participation.

## Author Contributions

RX and BY collected and analyzed the data and wrote the manuscript. CL and LM made substantial contributions planning this work. LL and LM substantially revised the work and manuscript. All authors listed have made a substantial, direct, and intellectual contribution to the work and approved it for publication.

## Funding

This work was supported by grants from the Natural Science Foundation of China (81972381 and 82173385).

## Conflict of Interest

The authors declare that the research was conducted in the absence of any commercial or financial relationships that could be construed as a potential conflict of interest.

## Publisher’s Note

All claims expressed in this article are solely those of the authors and do not necessarily represent those of their affiliated organizations, or those of the publisher, the editors and the reviewers. Any product that may be evaluated in this article, or claim that may be made by its manufacturer, is not guaranteed or endorsed by the publisher.
